# The LEAD trial - the effectiveness of a decision aid on decision making among citizens with lower educational attainment who have not participated in FIT-based colorectal cancer screening in Denmark: study protocol for a randomized controlled trial

**DOI:** 10.1186/s13063-018-2921-z

**Published:** 2018-10-10

**Authors:** Pernille Gabel, Mette Bach Larsen, Pia Kirkegaard, Adrian Edwards, Berit Andersen

**Affiliations:** 1 Department of Public Health Programmes, Randers Regional Hospital, Central Denmark Region, Skovlyvej 15, 8930 Randers NØ, Denmark; 2 Faculty of Health, Aarhus University, Aarhus, Denmark; 3 Division of Population Medicine, Cardiff University School of Medicine, Cardiff, UK

**Keywords:** Colorectal cancer, Cancer screening, Decision aid, Lower educational attainment, Randomized controlled trial

## Abstract

**Background:**

Colorectal cancer screening participation is a preference-sensitive choice, in which trade-offs between benefits and harms must be made by individual citizens. Often the decision is made without any contact with healthcare professionals. Citizens with lower educational attainment tend to participate less in colorectal cancer screening than citizens with average educational attainment. Further, they tend to have lower levels of knowledge about colorectal cancer screening. Providing lower educational attainment citizens with a targeted decision aid embracing their diverse information needs might increase these citizens’ ability to make informed decisions. The aim of this trial is to test the effectiveness of such a newly developed self-administered decision aid.

**Methods:**

The LEAD (Lower Educational Attainment Decision aid) trial will be conducted as a two-arm randomized controlled trial among 10,000 50–74-year-old citizens, resident in the Central Denmark Region not yet invited to take up colorectal cancer screening. Citizens will receive a baseline questionnaire. Respondents will be allocated into the intervention or the control groups. Citizens in the intervention group will receive the decision aid whereas the control group will not. Those who return a stool sample within 45 days after receiving the screening invitation and those with medium or higher educational attainment are excluded. Both groups will receive a follow-up questionnaire 90 days after being invited to colorectal cancer screening.

A historic cohort consisting of 5000 50–74-year-old citizens resident in the Central Denmark Region, having received their screening invitation in the beginning of 2017 will be included. This cohort will receive a follow-up questionnaire 6–9 months after they received the screening invitation.

Informed choice will be evaluated by assessing levels of knowledge, attitudes, and screening uptake. Analyses will be conducted as intention-to-treat analyses. Additionally, differences between levels of worry and decisional conflict between groups will be assessed as secondary outcomes.

**Discussion:**

This trial will evaluate whether a targeted decision aid is a feasible way of enhancing informed choice among lower educational attainment citizens in colorectal cancer screening. Further, it may guide decisions about providing information material in cancer screening in general.

**Trial registration:**

ClinicalTrials.gov, NCT03253888. Registered on 17 August 2017.

**Electronic supplementary material:**

The online version of this article (10.1186/s13063-018-2921-z) contains supplementary material, which is available to authorized users.

## Background

Colorectal cancer (CRC) is a common cause of cancer-related deaths worldwide. In developed countries the age-standardized mortality rate of CRC is 14.7 and 9.3 per 100,000 men and women, respectively [1]. However, CRC mortality can be reduced by 25% in citizens undergoing screening with the guaiac fecal occult blood test (gFOBT) at least once [2]. The fecal immunochemical test (FIT) is superior to the gFOBT in detecting CRC and is now the preferred test [3].

Screening has obvious benefits, including reduced incidence of CRC, earlier stage diagnoses, and reduced mortality [2]. However, there are also adverse effects of screening, such as risk of over-diagnosis, unnecessary tests and complications to these, and psychological consequences. Hence, a decision to take up screening is preference-sensitive and includes a cognitive and emotional trade-off between benefits and harms, based on adequate information [4]. From an ethical standpoint, such a decision should be an informed decision.

In order to make an informed decision, comprehensible and easily accessible information is crucial. However, information material about CRC screening is often neither read nor understood by citizens with lower levels of health literacy [5]. Low health literacy is significantly associated with lower educational attainment [6]. In order to support informed decision making, decision aids have been developed to provide balanced information on healthcare [7]. Decision aids come in different formats (e.g. booklet, leaflet, online service, DVD, etc.) and they may encourage readers to reflect on the information in a structured way, for example by the means of “values clarification exercises” [8]. Studies have shown that decision aids can increase CRC knowledge and the proportion of citizens making an informed decision about screening uptake when used by citizens and healthcare professionals together [9, 10]. Furthermore, they can increase screening uptake [11–15]. However, the decision to take up CRC screening is often made without prior contact with healthcare professionals, and hence, decision aids need to be self-administered. Self-administered decision aids increase CRC knowledge [16–20] and informed choices made [18, 19], but may result in more negative screening attitudes [18, 19], while studies regarding screening uptake are inconclusive [16, 18–20].

In general, lower educational attainment citizens participate less in CRC screening compared with those who have average educational attainment [21, 22]. Further, they have higher CRC mortality compared to medium and higher educational attainment citizens [23, 24]. Tailoring a decision aid to this group could be advantageous, but their information needs are diverse, ranging from a desire for a clear recommendation to the need to know all the details [25]. Decision aids tailored to this group do exist [9, 10, 13], but are not self-administered [19], and none of them embrace diverse information and support needs. Hence, this research group has developed a decision aid tailored for lower educational attainment citizens [26]. It was designed as an online format, presenting information in steps, making it possible for citizens to get as much or as little information as they want, thereby embracing diverse information needs.

The aim of the LEAD (Lower Educational Attainment Decision aid) trial is to investigate the effectiveness of the newly developed decision aid tailored for lower educational attainment citizens on informed choice, as assessed based on knowledge of CRC and CRC screening, CRC screening uptake, and CRC screening attitudes. Further, the effectiveness on levels of CRC worries and decisional conflict will be assessed.

## Methods

### Setting

CRC screening with biennial FIT was introduced in Denmark in 2014 with an initial “prevalence round” of invitations for four years, where all citizens aged 50–74 years are invited once, according to month of birth. Citizens turning 50 or 75 years during the prevalence round are invited just before their birthday if not invited earlier. From 2018, all citizens in the target group will receive biennial invitations (identifying incident CRC). The screening program is locally administered in five regions. This study is carried out in the Central Denmark Region, which is the second largest region with roughly 395,000 citizens aged 50–74 years [27].

In the CRC screening program, the mailed screening invitation contains a screening kit (collection tube and collection paper), instructions on how to obtain the sample, an official information leaflet, and a pre-stamped, pre-addressed envelope. If a screening sample is not returned within 45 days, a digital reminder is sent (see below). Test results are digitally mailed to the citizens within one week after the test is received at the laboratory. Citizens with a negative test result are referred to the next screening round (two-year interval) and citizens with a positive test result receive an appointment for colonoscopy. Non-participating citizens are automatically referred to the next screening round.

Danish residents are obliged to order a digital signature which is used to log in to a secure national e-mail platform, where all mandatory digital communication with Danish authorities (including hospitals) takes place via the unique civil registration number. Some citizens (8.7% of the population aged 45–74 years during the summer of 2017 [28]) can be exempted from digital communication [29] and receive conventional mail via a remote printing system, in which an external supplier prints out and sends all letters to exempted citizens.

### Study design

**Fig. 1:**
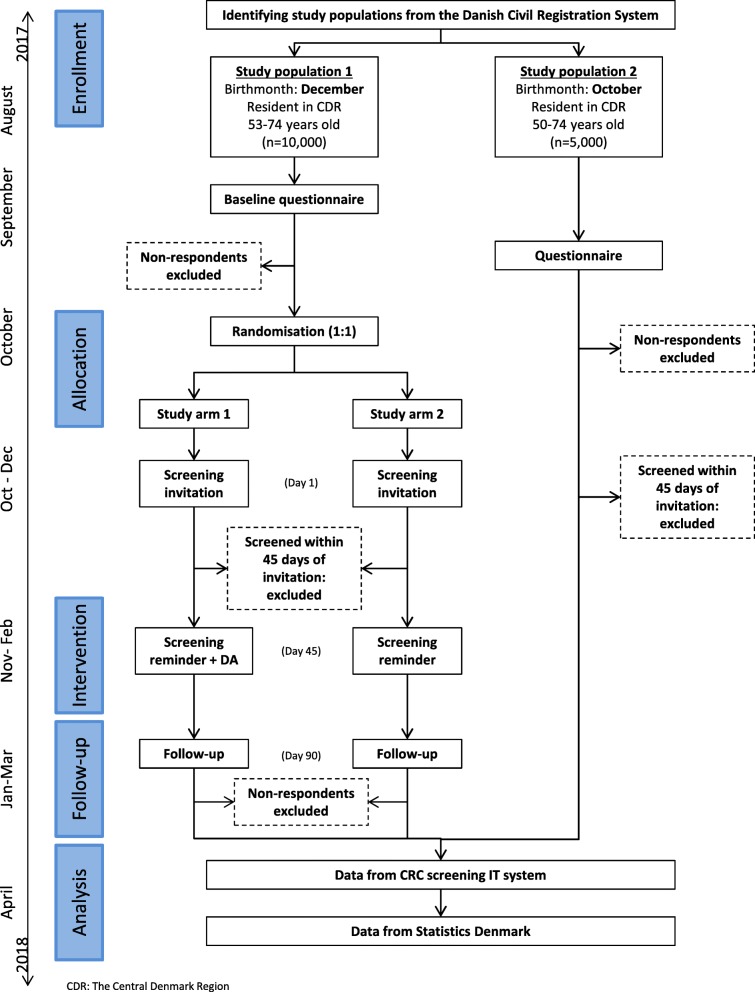
Participant *flow chart*. Figure depicting the data collection, interventions, and participant flow in the trial

The LEAD trial will be conducted as a two-armed, randomized controlled trial nested into the Danish CRC screening program in the Central Denmark Region. Included citizens will be randomly allocated to one of the following two study arms (Fig. 1):

1. Screening reminder with the decision aid (intervention group);
2. Screening reminder without decision aid (usual care/control group).

The intervention and control groups receive the baseline questionnaire before the screening invitation. This might increase screening awareness and possibly screening uptake and knowledge in these groups – a true Hawthorne effect. To be able to assess the effect of the questionnaire, a historic cohort will be introduced as in other decision aid trials [30]. This group will receive their questionnaire after screening invitation and uptake assessment.

This study entails evaluating the offer to use the decision aid. Even though participants may not necessarily use it, not using the decision aid is not a specific issue in this study as it is a pragmatic evaluation of offering it in a routine screening program.

#### Study processes

Seven to 20 weeks before the invitation date, the included citizens in study arms 1 and 2 will receive a link to the baseline questionnaire via their digital mail (with remote printing for exempted citizens). Questionnaire reminders will be sent out after two weeks. Instead of a second questionnaire reminder, non-respondents will be contacted via telephone after four weeks and invited to fill out the questionnaire via telephone interview. In a pilot study, the response rates among lower educational attainment and medium/higher educational attainment citizens using an invitation and a two-week reminder were assessed at 27% and 55%, respectively. The response rates increased to 40% and 64%, respectively, in the two groups, by using telephone interviews. Accordingly, we expect telephone interviews to help reach more of the lower educational attainment citizens. Questionnaire respondents will also be asked for consent to the obtaining and analysis of screening data (date of invitation, reminder, receipt of test in laboratory, and test result) from the screening program database.

Citizens who respond to the questionnaire and give consent to the use of registry data will be randomly allocated into study arms 1 and 2. Allocation will be performed in the ratio 1:1 and will use a computer-generated algorithm for randomization, based on a simple randomization procedure. Randomization will be conducted based on the study participants’ record-ID numbers. Participants will not be able to change study arm after allocation and there will be no blinding.

Citizens who take up screening within 45 days after the screening invitation and citizens with medium or higher educational attainment (i.e. 10 years or more of education) will be excluded from the analyses after randomization. This might cause attrition bias. However, randomization will be conducted externally before screening uptake is known. Furthermore, educational attainment will not be available to researchers at an individual level, unless data are pseudonymized. However, educational level and screening uptake within 45 days of invitation is not expected to be associated with study arm and, hence, no attrition bias is expected.

When citizens in the intervention group are due for a screening reminder, they will concurrently receive a separate mail containing a link for the decision aid. This strategy was chosen in order to avoid information overload, since other conventional information material is sent out along with the screening invitation. Further, first invitations are sent out by postal mail, and hence, the link is better provided in the reminders that are sent by digital mail.

Forty-five days after the citizens have received the intervention/screening reminder, they will receive the follow-up questionnaire. Reminder procedures for the follow-up questionnaire will be the same as for the baseline questionnaire. Citizens who do not return follow-up questionnaires within six weeks (i.e. going to the last page in the questionnaire and pressing submit) will be excluded from the study, since the consent to use data in the project will not have been given.

Citizens in the historic study arm will receive one questionnaire 6–8 months after being invited to participate in CRC screening. Reminder procedures for this questionnaire will be the same as for the previous questionnaires.

#### Decision aid

The development of the decision aid was based on a framework suggested by Coulter et al. [31], in accordance with the IPDASi (the International Patient Decision Aid Standards instrument) criteria [32]. The process comprised six steps: (1) defining the purpose of the decision aid and the target population, i.e. decision about CRC screening uptake in lower educational attainment citizens; (2) formation of steering group, comprising both screening experts and experts on shared decision making (no citizens were included in the steering group); (3) designing the decision aid, based on Danish lower educational attainment citizens’ information needs, as described in a previous study [25]; (4) alpha testing the decision aid to check usability and design – this step included both lower educational attainment citizens and healthcare professionals and used both focus group interviews and e-mail correspondence; (5) external peer reviewing also included citizens and healthcare professionals; and (6) the beta or user-testing with lower educational attainment citizens assessed feasibility, comprehensibility, and usability. Based on inputs from all steps, the final decision aid was completed by a professional web design agency, supervised by the steering group [26].

The decision aid is an online tool. The online design was chosen, since digital communication is mandatory in Denmark, and 91.3% of the population (aged 45–74 years) has access to digital mail [28]. Further, the online design opens up possibilities to feature more advanced functions, e.g. questions for the participant during the process, and a summary of answers at the end, presented as a “choice indicator.” The decision aid consists of 15 steps in total. Each step presents facts in a figure or chart and, via links in these, pop-ups with further information are available. The figures used are icons (e.g. illustrating colon with a polyp and cancer), pie charts, crowd-figures, and a flow chart illustrating the possible steps in CRC screening. Almost all pop-ups have a “read more” function. The decision aid is available in Danish by contacting the authors.

### Study population

Two samples of citizens will be provided by the Danish Health Data Authority from the Danish Civil Registration System [33]: (1) a random sample of 10,000 53–74-year-old citizens, born in December, and resident in the Central Denmark region on 1 August 2017 (expected to be invited for screening during October–December 2017); and (2) a random sample of 5000 50–74-year-old citizens, born in October, and resident in the Central Denmark Region on 1 August 2017 (invited for screening during January–March 2017) for the historic study arm (Fig. 1). The lists will contain civil registration number, name, and postal address of the sampled citizens. No incentives are used in this trial.

### Outcomes

The outcomes assessed in this trial are presented in Table 1.

**Table 1:** Trial outcomes

Outcome	Characteristics	Assessment time	Comparisons^a^	Null Hypothesis^a^
Primary outcome (Informed choice elements)				
Knowledge	Numeric scale	Baseline	1 vs. 2	No difference
	Numeric scale	Dif_Follow-up-baseline_^b^	1 vs. 2	1 > 2
	Numeric scale	Follow-up	1 vs. 2 vs H	1 > 2 and 1 > H
Attitudes	Numeric scale	Baseline	1 vs. 2	No difference
	Numeric scale	Dif_Follow-up-baseline_^b^	1 vs. 2	No difference
Uptake	Dichotomous	Follow-up	1 vs. 2 vs H	1 > 2 and 1 > H
Secondary outcomes				
Decisional conflict	Numeric scale	Follow-up	1 vs. 2 vs H	1 < 2 and 1 < H
Worry	Numeric scale	Baseline	1 vs. 2 vs H	No difference
	Numeric scale	Dif_Follow-up-Baseline_^b^	1 vs 2	1 < 2
Effect modifiers and confounders				
Health literacy	Numeric scale	Baseline	1 vs. 2 vs. H	No difference
Background data				
Family income	Categorical	Post survey	1 vs. 2 vs. H	No difference
Occupation	Categorical	Post survey	1 vs. 2 vs. H	No difference
Marital status	Dichotomous	Post survey	1 vs. 2 vs. H	No difference
Ethnicity	Categorical	Post survey	1 vs. 2 vs. H	No difference

*H* historic study arm

^a^Group 1 refers to the intervention group and group 2 refers to the control group

^b^Dif_Follow-up-Baseline_: The mean increase or decrease in knowledge, attitude, and worry-score from baseline to follow-up will be estimated. Means will be compared between the two study arms

#### Primary outcome

Informed choice will be evaluated based on the following primary outcomes: consistency of knowledge about CRC screening, attitudes towards CRC screening, and screening uptake [34].

Knowledge will be measured via questionnaire before and after intervention using a seven-item scale developed by the authors based on both a literature search and patient information needs as previously clarified in focus group interviews [25]. Post-intervention levels and changes from baseline to follow-up will be evaluated.

Screening attitudes will be estimated using the translated four-item attitudes scale, ranging from 4 to 28 points [34]. The scale has been translated into Danish from English with conventional forward-backward translation [35]. Attitudes will be assessed at baseline and at follow-up.

Data on screening uptake will be collected from the CRC screening program database. Citizens will be defined as having taken up CRC screening if they return a stool sample within three months (90 days) after the screening invitation has been sent out [36].

#### Secondary outcomes

The 16-item decisional conflict scale [37] will be used to measure decisional conflict, i.e. uncertainty about making the right choice. It comprises five subscales, including the decisional support subscale and the (perceived) effectiveness of the decision subscale. Scores range from 0 to 100 and data are collected at follow-up.

Worry about CRC will be assessed using three items on worry about CRC. Two items assess worry and anxiety when considering CRC screening and one assesses worry about the result of a screening test. Items have also been developed by the authors based on literature searches [19, 20, 38–40]. Scores are in the range of 3–15. Assessments are made at baseline and follow-up.

Health literacy is a multidimensional measure of a person’s ability to access, understand, appraise, and apply information about healthcare, disease prevention, and health promotion [41]. It will be measured at baseline using the 16-item HLS-EU-Q16 scale [41].

The use of the decision aid will be measured among citizens receiving an invitation to use it. However, detailed process data will not be collected, since the primary aim of this trial is to investigate the effectiveness of having the decision aid provided, regardless of actually reading it. Usability and acceptability have been further assessed in the development phase of the decision aid [26].

#### Background data

Background data on participants’ sociodemographic and socioeconomic characteristics will be obtained from Statistics Denmark [42] by the end of the study period. These data are updated annually.

Lower educational attainment is defined according to the UNESCO classification of basic education as < 10 years of education [43].

Family income will be divided into three categories based on lower, middle, and upper tertiles of the dataset. Occupation is a categorical variable with the following six categories: Employed; Self-employed/chief executive; Unemployed/receiving benefits; Retired; Social welfare recipients; and Others. Marital status is dichotomized into two categories: married/cohabitant and single. Ethnicity will be a categorical variable with three categories: Danish; Western immigrant (Western Europe and North America); and non-Western immigrant (Table 1).

#### Questionnaires

**Fig. 2:**
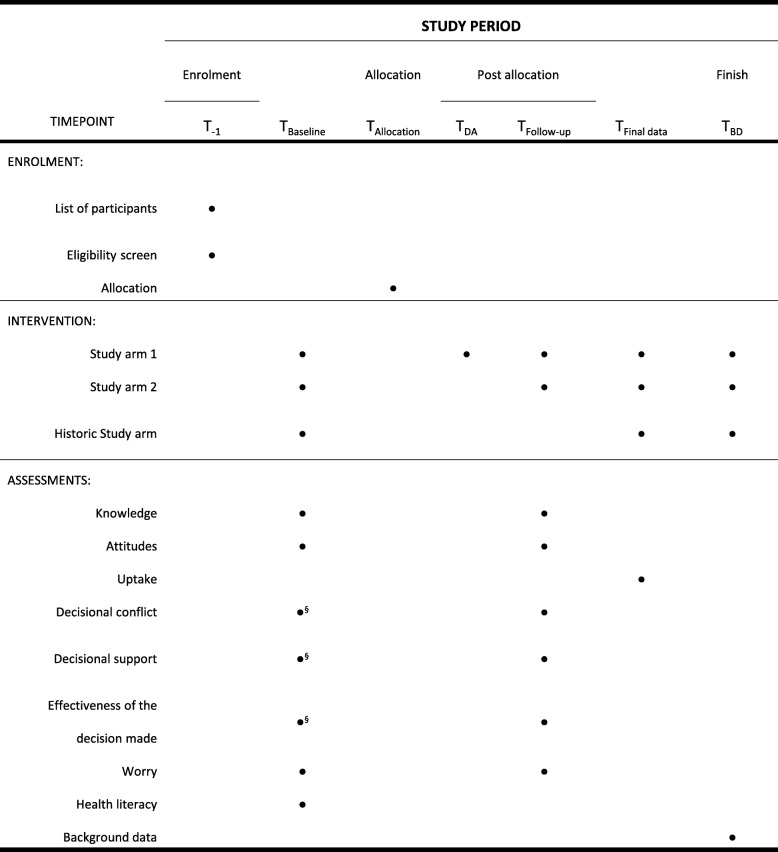
Schedule of enrolment, intervention, and assessments. Figure depicting the enrolment, intervention, and assessments according to SPIRIT 2013 statement [46]. ^§^ Assessed only in the historic cohort. BD background data, obtained from Statistics Denmark. Follow-up occurs throughout a period of 12 weeks. The final data will be obtained from the screening program IT system when all follow-up data have been collected. The complete data will then be merged with background data from Statistics Denmark

Three different questionnaires will be administered in this trial: the baseline (knowledge, seven items; attitudes, four items [34]; worry, three items; health literacy – HLS-EU-Q16, 16 items [41, 44]) and follow-up (knowledge; attitudes; worry; decisional conflict, 16 items [37]) questionnaires, and the questionnaire for the historic study arm participants (knowledge; attitudes; worry; health literacy; decisional conflict) (Fig. 2).

All questionnaires will be administered via REDCap Software (Version 6.12.0 - © 2016 Vanderbilt University) [45]. Data entry will occur automatically when the citizens complete the electronic questionnaires, hence no manual data entry will be required. The questionnaires are presented in Additional files 1, 2 and 3.

### Statistical methods

All statistical analyses will be carried out in Stata/SE 14 (STATACorp LP, College Station, TX, USA). Analyses will be carried out on a 5% significance level, stratified to gender. Estimates will be presented with 95% confidence intervals. Intention-to-treat analyses will be conducted.

Pearson’s chi-square test will be used to test differences in demographic characteristics between respondents and non-respondents as well as between those included in each study arm with lower educational attainment, in order to test for baseline differences. Furthermore, differences in educational attainment and screening uptake within 45 days in each study arm will be compared in order to evaluate the risk of attrition bias.

The main outcome of the trial is informed choice. Informed choice is difficult to measure and the evaluation will be based on adequate knowledge about CRC screening and consistency between attitudes towards CRC screening and screening uptake. This will be judged at group level not individual level. [46] Differences in distributions of responses to knowledge, attitudes, uptake, decisional conflict, and worries in the study arms will be analyzed using the independent sample t-test for continuous variables and Pearson’s chi-square test for categorical variables. The relative risk (RR) will be estimated for uptake using a logistic regression analysis with study arm 2 as the reference comparing to both study arm 1 and the historic cohort. Citizens are defined as having taken up CRC screening if they returned a stool sample within three months after the screening invitation was sent out [47]. For the numeric scale outcomes, a linear regression analysis will be conducted to compare the means between study arms 1 and 2 and study arm 2 and the historic cohort, using study arm 2 as the reference. For ordinal scales, not normally distributed as assessed by histograms and qq-plots, ordinal regression analyses will be conducted, estimating odds ratios for higher scores in study arm 1 and the historic cohort as compared to study arm 2, using study arm 2 as the reference.

Further, generalized linear models from the binary family will be conducted to test if socioeconomic variables (including health literacy) modify the effectiveness of the decision aid on knowledge, attitudes, and uptake. *P* values for these analyses will be estimated using the Wald test.

Few missing data are expected in registry and IT system data, since these systems are almost 100% complete [48]. Missing data may occur with the questionnaire data. In the scoring of health literacy, no more than three missing out of 16 items is accepted, otherwise the score cannot be calculated [41]. This is in contrast to the decisional conflict scale, in which the number of missing items is considered when calculating the final score [37]. In the knowledge scale, all questions are multiple choices and the response category “I don’t know” is represented in every item, giving the respondents a possibility to answer even if they are in doubt. The answer “I don’t know” and missing values are coded as wrong answers in the final scoring.

### Power calculations

The smallest difference between outcomes between groups is expected to be found in attitudes estimates (a 14% difference in proportions having a positive attitude towards screening [19]) Based on this assumption, power calculations (considering a 5% significance level and an 80% statistical power) indicate that we need to include 200 citizens with lower educational attainment in each group. We expect 47% of citizens invited to CRC screening not to return a stool sample within 45 days. Further, we know that 26% of the population has lower educational attainment [49]. By combining mailed invitations and reminders with telephone calls, we aim to reach a response rate of at least 60% for baseline questionnaire and 80% for follow-up (i.e. study participants). Further, we expect a 50% response rate in the historic study arm. In order to be able to make gender stratified analyses, and considering the rate of screening reminder, rate of lower educational attainment, and questionnaire response rate, we need to include 5000 citizens in each study arm.

## Discussion

The decision to take up CRC cancer screening is a preference-sensitive choice. The decision is often made without prior contact with healthcare professionals, and hence, comprehensible and accessible information material is crucial. Currently this type of information material is lacking, especially for lower educational attainment citizens, who also have the lowest screening uptake. Our newly developed decision aid is designed to provide lower educational attainment citizens with accessible and comprehensible information to support informed choice about taking up screening. Further, the decision aid is easy to implement directly into the existing screening program, through the national electronic mail system, should it be shown effective on our key outcomes. Preferably, all decisions about screening uptake and non-uptake should be informed. In this trial, we investigate whether non-uptake is informed and can be supported. However, the follow-up questionnaire will be sent out to screening participants as well as non-participants. In a future study, it will be possible to investigate whether screening uptake was informed. By nesting the effectiveness study into an existing screening program using registry data of high quality and with almost 100% coverage [48], the results of this study, combined with future information about the level of informed choices of uptake and non-uptake, may provide a basis for recommendations about targeted information material in CRC screening in particular and guide future information and decision support material in cancer screening in general.

## Additional files

Additional file 1: Baseline questionnaire. (DOCX 37 kb)

Additional file 2: Follow-up questionnaire. (DOCX 39 kb)

Additional file 3: Questionnaire for the historic cohort. (DOCX 37 kb)

Additional file 4: Trial registration. (DOCX 38 kb)

## Abbreviations

CRC: Colorectal cancer
FIT: Fecal immunochemical test
gFOBT: Guaiac fecal occult blood test
IPDASi: International patient decision aid standards collaboration instrument
LEAD trial: Lower educational attainment decision aid trial
RR: Relative risk
