# Supplementary material for: The LEAD trial - the effectiveness of a decision aid on decision making among citizens with lower educational attainment who have not participated in FIT-based colorectal cancer screening in Denmark: study protocol for a randomized controlled trial
Source: Trials. 2018 Oct 10;19:543. doi: 10.1186/s13063-018-2921-z (PMC6180588; doi:10.1186/s13063-018-2921-z)
Supplement: Supplementary file 2 — Follow-up questionnaire. (DOCX 39 kb) [file 13063_2018_2921_MOESM2_ESM.docx]

# Additional file 2 – Follow-up questionnaire

Thank you for filling out yet another questionnaire.

The questionnaire consists of 4 pages. Each page contains 3 – 16 questions, to which we kindly ask you to respond. You will see that some of the questions are identical to those posed in former questionnaire; this is quite intended. Other questions are new.

**Thank you for your cooperation.**

| Below you will find 7 statements. Please respond based on your actual knowledge without finding the answers on the internet or similar. | | | | | |
| --- | --- | --- | --- | --- | --- |
| 1. Colorectal cancer is often triggered by a scratch in rectum | Right  □ |  | Wrong  □ |  | I don't know  □ |
| 1. 1 out of 20-25 people will be diagnosed with colorectal cancer before the age of 75 years | Right  □ |  | Wrong  □ |  | I don't know  □ |
| 1. It is possible to have an undetected colorectal cancer for a longer period of time without having any symptoms | Right  □ |  | Wrong  □ |  | I don't know  □ |
| 1. Only people having symptoms of colorectal cancer can participate in screening | Right  □ |  | Wrong  □ |  | I don't know  □ |
| 1. You will have to go to the doctor, if you have symptoms of colorectal cancer, although the screening result did not detect any blood in the stool | Right  □ |  | Wrong  □ |  | I don't know  □ |
| 1. Blood in the stool is an obvious sign of colorectal cancer | Right  □ |  | Wrong  □ |  | I don't know  □ |
| 1. Abdominal pain and altered bowel habits may be symptoms of colorectal cancer | Right  □ |  | Wrong  □ |  | I don't know  □ |

| The below 3 questions concern your thoughts about colorectal cancer | | | | | |
| --- | --- | --- | --- | --- | --- |
| 1. I become worried when I think about colorectal cancer | Totally disagree  □ | Disgree  □ | Neither agree nor disagree  □ | Agree  □ | Fully agree  □ |
| 1. I become scared when I think about colorectal cancer | Totally disagree  □ | Disgree  □ | Neither agree nor disagree  □ | Agree  □ | Fully agree  □ |
| 1. I am concerned that colorectal cancer is detected if I participate in screening | Totally disagree  □ | Disgree  □ | Neither agree nor disagree  □ | Agree  □ | Fully agree  □ |

Questions 11-14 comprise the four items of the attitudes scale presented by Marteau et al [34].

Questions 15-30 comprise the 16 items of the decisional conflict scale [37].

Citizens receiving the decision aid:

| You have received a link for an alternative information material regarding bowel cancer screening.  The last questions are about this information material. | | | | | |
| --- | --- | --- | --- | --- | --- |
| 1. Did you use the link you received? | Yes  □ |  | No  □ |  |  |
| 1. If you have any comments regarding the information material, please feel free to provide them here: | ________________________________________________________________ | | | | |
| 1. If you have any comments regarding the questionnaire, please feel free to provide them here: | ________________________________________________________________ | | | | |

Citizens not receiving the decision aid:

| In this page, you have the opportunity to comment on the questionnaire | |
| --- | --- |
| 1. If you have any comments regarding the questionnaire, please feel free to provide them here: | ________________________________________________________________ |

**When you have completed the questionnaire, please press "Submit".**

-
