# Supplementary material for: The LEAD trial - the effectiveness of a decision aid on decision making among citizens with lower educational attainment who have not participated in FIT-based colorectal cancer screening in Denmark: study protocol for a randomized controlled trial
Source: Trials. 2018 Oct 10;19:543. doi: 10.1186/s13063-018-2921-z (PMC6180588; doi:10.1186/s13063-018-2921-z)
Supplement: Supplementary file 4 — Trial registration. (DOCX 38 kb) [file 13063_2018_2921_MOESM4_ESM.docx]

# Additional file 4 – Trial registration

| Data category | Information |
| --- | --- |
| Primary registry and trial identifying number | ClinicalTrials.gov NCT03253888 |
| Date of registration in primary registry | August 17 2017 |
| Secondary identifying numbers | The Central Denmark Region Committees on Health Research Ethics (j. no.: 143/2016)  The Danish Data Protection Agency (J. no.: 2012-58-006/Case number: 1-16-02-94-16)  The Danish Patient Safety Authorities (J. no.: 3-313-1729-1) |
| Source(s) of monetary or material support | The Danish Foundation TrygFonden  The Danish Cancer society  The Health Research Fund of Central Denmark Region  Health, Aarhus University, Denmark  Danish Cancer Research Foundation  The Health Foundation, Denmark  The Private Foundation of the Family Spogaard  The Private Foundation of Ringgaard-Bohn  (The Danish Health and Medicine Authorities) |
| Primary sponsor | The Danish Foundation TrygFonden |
| Secondary sponsor(s) | The Danish Cancer Society, The Central Denmark Region, Aarhus University, Danish Cancer Research Foundation, The Health Foundation (Denmark), and private funds |
| Contact for public queries | Pernille Gabel, MD, [pergab@rm.dk](mailto:pergab@rm.dk) |
| Contact for scientific queries | Pernille Gabel, MD, Department of Public Health Programmes, Randers Regional Hospital, Randers, Denmark |
| Public title | The effectiveness of a decision aid in lower educational attainment bowel cancer screening non participants on informed choice (LEAD) |
| Scientific title | The LEAD study: the effectiveness of a decision aid in lower educational attainment colorectal cancer screening non participants in the central Denmark Region – a randomised controlled trial |
| Countries of recruitment | Denmark (The Central Denmark Region) |
| Health condition(s) or problem(s) studied | Decision aid, knowledge, attitudes toward CRC screening, uptake |
| Intervention(s) | Decision aid offered along with colorectal cancer screening reminder |
|  | Colorectal cancer screening reminder without decision aid |
| Key inclusion and exclusion criteria | Ages eligible: ≥50 years and <75 years of age |
|  | Sexes eligible: both men and women |
|  | Inclusion criteria: residents of the Central Denmark Region with month of birth in December (Study arm 1 and 2) or October (Historic cohort) |
|  | Exclusion criteria: Does not receive an invitation for colorectal cancer screening during the study period or takes up colorectal cancer screening within 45 days of the invitation or more than 10 years of education |
| Study type | Interventional |
|  | Allocation: randomised; Intervention model: parallel assignment |
|  | Primary purpose: increased level of informed choice in colorectal cancer screening |
|  | Phase III |
| Date of first enrolment | August 14 2017 |
| Target sample size | 4,620 |
| Recruitment status | Ongoing |
| Primary outcome(s) | Knowledge, attitudes and uptake (informed choice) |
| Key secondary outcome(s) | Worry about colorectal cancer |
|  | Decisional conflict |
|  | Decisional support |
|  | Effectiveness of the decision made |
